# Stem Cells Therapy for Spinal Cord Injury

**DOI:** 10.3390/ijms19041039

**Published:** 2018-03-30

**Authors:** Marina Gazdic, Vladislav Volarevic, C. Randall Harrell, Crissy Fellabaum, Nemanja Jovicic, Nebojsa Arsenijevic, Miodrag Stojkovic

**Affiliations:** 1 Faculty of Medical Sciences, Department of Genetics, University of Kragujevac, 34000 Kragujevac, Serbia; marinagazdic87@gmail.com; 2 Faculty of Medical Sciences, Department of Microbiology and immunology, Center for Molecular Medicine and Stem Cell Research, University of Kragujevac, 34000 Kragujevac, Serbia; drvolarevic@yahoo.com (V.V.); arne@medf.kg.ac.rs (N.A.); 3 Regenerative Processing Plant, LLC, 34176 US Highway 19 N Palm Harbor, Palm Harbor, FL 34684, USA; dr.harrell@regenerativeplant.org (C.R.H.); crissy@regenerativeplant.org (C.F.); 4 Faculty of Medical Sciences, Department of Histology and embryology, University of Kragujevac, 34000 Kragujevac, Serbia; nemanjajovicic.kg@gmail.com; 5 Spebo Medical, 16 Norvezanska, 16000 Leskovac, Serbia

**Keywords:** spinal cord injury, stem cells, embryonic stem cells, induced pluripotent stem cells, ependymal stem/progenitor cells

## Abstract

Spinal cord injury (SCI), a serious public health issue, most likely occurs in previously healthy young adults. Current therapeutic strategies for SCI includes surgical decompression and pharmacotherapy, however, there is still no gold standard for the treatment of this devastating condition. Inefficiency and adverse effects of standard therapy indicate that novel therapeutic strategies are required. Because of their neuroregenerative and neuroprotective properties, stem cells are a promising tool for the treatment of SCI. Herein, we summarize and discuss the promising therapeutic potential of human embryonic stem cells (hESC), induced pluripotent stem cells (iPSC) and ependymal stem/progenitor cells (epSPC) for SCI.

## 1. Introduction

Spinal cord injury (SCI), a serious public health issue, most likely occurs in previously healthy young adults. Country-level incidence studies show that incidenceof SCI ranges from 40 to 80 per million people per year [1]. The aim of modern society is to prevent traumatic SCI usually caused by road traffic accidents, falls from heights, and violence (gunshot or stab wound), as well as nontraumatic SCI resulting from cancer, spinal disk degeneration, or arthritis. Gray and white matter damage after SCI leads to partial or complete motor, sensory, or autonomic deficit in parts of the body distal to the lesion site. In accordance, the most devastating of all SCIs are injuries of the cervical spine, accompanied by high-grade dysfunction of the central nervous system (CNS). In order to determine the severity of SCI, the American Spinal Injury Association (ASIA) defined international standards for neurological classification and formulated impairment scale for neurological assessment of individuals with SCI [2]. The most severe SCI is complete, irreversible and characterized by increased mortality risk compared to the general population due to cardiovascular, respiratory and genitourinary complications, deep venous thrombosis, chronic neuropathic pain, pressure ulcers, and infections [3].

Although a detailed understanding of molecular mechanisms involved in the pathophysiology of SCI provides great promise for improving therapeutic strategies for spinal cord repair, there is still no gold standard for the treatment of this devastating condition. Because of their differentiation abilities and secretion of a variety of cytokines and growth factors, stem cells have been extensively studied as a novel neuroregenerative and neuroprotective agents for the treatment of SCI. A large number of murine models of acute, subacute and chronic SCI, that resemble lesions that develop in the adult human spinal cord following exposure to trauma, have demonstrated not only therapeutic role of in vitro-expanded stem cell-derived progenitor, but also the cellular and molecular mechanism of spinal cord repair and neurological improvements through activation of endogenous stem cells.

In this review, we summarize the advantages and disadvantages of current surgical and pharmacological approaches and discuss promising therapeutic potential of human embryonic stem cells (hESC), induced pluripotent stem cells (iPSC) and ependymal stem/progenitor cells (epSPC) for SCI. We hope that the data highlighted in this report may be of relevance to stem cell researchers and clinicians as a backdrop to future development of stem cell-based therapy of SCI.

## 2. Pathophysiology

Neurological outcome after SCI is associated with mechanical destruction of spinal tissue and secondary injury mediated by multiple pathophysiological processes (reviewed in [4]). Displacement of the anatomical structures of the spinal cord, following the initial mechanical events underlies the onset of SCI and refers to the primary injury phase. Physical forces such as compression, contusion, laceration, and acute stretch, damage nerve cells and their axons, leading to the disruption of descending neuronal pathways [5]. During the secondary phase of SCI, the series of destructive pathological processes occur, leading to massive cellular dysfunction and death, aberrant molecular signaling and generation of harmful metabolic products. Immediately after sudden mechanical trauma, focal microhemorrhage, vasospasm and reduction in blood flow, are seen within the injured cord. Disturbance in ion chomeostasis, glutamaterelease, and lipid peroxidation in the lesion site contribute to further progression of neurological dysfunction in patients with SCI by activating consequential cascade of destructive pathophysiological mechanisms [6,7,8]. Electrolytic disbalance in SCI is characterized by elevated extracellular concentration of potassium (K^+^) and increased intracellular concentration of sodium (Na^+^) and calcium (Ca^2+^) [9]. As a result of the high amount of potassium in the extracellular area, the transmission of a nerve impulse is blocked. Intracellular acidosis promotes excessive water influx into the neurons, resulting in cytotoxic edema and neuronal death (reviewed in [10,11]. Damaged cells massively release various toxic metabolites as well as excitatory amino acid glutamate, which trigger autodestructive free-radical generation and excitotoxicity [9,12]. Reactive oxygen species cause oxidative damage of DNA, and lipid peroxidation in the cellular membranes. These pathological changes observed in the lesion site lead to further progression of neurons and glia cells necrosis and apoptosis [13]. It is well known that immune response significantly contributes to pathogenesis of SCI. Interestingly, the inflammatory process has both aggressive and protective effects on damaged spinal cord. Mechanical trauma breaks the integrity and increases permeability of the blood-spinal cord barrier, thus contributing to inflammatory cell invasion, and edema generation at the lesion site. Neutrophils, macrophages, T cells and microglia infiltrate the spinal parenchyma, and produce wide range of proinflammatory cytokines tumor necrosis factor alpha (TNF-α), interleukin-1 beta (IL-1β), interleukin-1 alpha (IL-1α), and interleukin-6 (IL-6) [14,15,16,17]. Expression of ion channels included in the family of connexins (Cx) is augmented at early stages after traumatic SCI and contributes to secondary damage of spinal cord and neuropathic pain [18]. Neuroinflammation causes the development of a necrotic cavity surrounded by a glial scar that prevents SCI progression [19]. At the same time, activated macrophages and microglia are involved in phagocytosis of necrotic and destroyed tissue. This rapid removal of cellular debris is significant for establishing an environment beneficial for neuroregeneration [20,21].

## 3. Current Therapeutic Strategies for Spinal Cord Injury

### 3.1. Surgical Decompression

Surgical Timing in Acute Spinal Cord Injury Study (STASCIS) showed that urgent surgical decompression within 24 h after injury significantly increased post-operative motor and sensory functions according to American Spinal Injury Association (ASIA) score [22,23]. Although early decompression reduces the risk of respiratory failure and sepsis [24], neurological surgery for the treatment of SCI is still associated with complications such as incidental durotomy or meningitis [25]. These limitations indicate that STASCIS and urgent neurological surgery in SCI as a widely adopted treatment requires further improvement.

### 3.2. Therapeutic Hypothermia

Beneficial effects of modest (32–34 °C) systemic hypothermia induced by intravascular cooling catheter have been clearly demonstrated in SCI [26,27]. Problems related to the safety of invasive systemic treatment and relation between temperature at the spinal cord lesion and core body temperature have been successfully resolved by localized cooling of the injury site during surgical decompression [28]. Systemic hypothermia as well as local cooling attenuate main pathophysiological processes during SCI including neuronal metabolism, neuroinflammation, oxidative stress, excitotoxicity, and apoptosis [29,30]. At the same time, decreased temperature protects the spinal cord from further injury by preserving the blood-spinal cord barrier (BSCB) and reducing edema, and induces neurorepair by enhancing angiogenesis and neurogenesis [29]. However, the effects of timing and duration of hypothermia as well as the best rewarming method on endogenous mechanisms and mobilization of stem cells are still the main challenges in the use of therapeutic hypothermia for the treatment of SCI [31].

### 3.3. Pharmacotherapy

Because of their anti-inflammatory effects and capacity to reduce oxidative stress and excitotoxicity, corticosteroids are used in many preclinical and clinical studies as therapeutic agents for the treatment of SCI [32]. Although it was expected that methylprednisolone would have the ability to suppress immune-mediated damage after SCI by decreasing the inflammatory cytokine production, several clinical studies failed to demonstrate functional repair after methylprednisolone administration [33,34,35,36,37]. Use of methylprednisolone in patients with SCI has become controversial, because of high rate of complications such as sepsis, pulmonary embolism, and gastrointestinal hemorrhage [33,34,35,36,37]. In order to improve pharmacological treatment of secondary injury phase, neuroprotective and regenerative effects of various agents were evaluated in clinical trials during the last decade. Several prospective, multicenter human studies demonstrated neurologic improvement in patients with spinal cord trauma who received riluzole [38], GM1 ganglioside [39], BA-210 [40], minocycline [41] or granulocyte-colony stimulating factor (G-CSF) [42], suggesting the potential of these agents for application in routine clinical practice.

## 4. Stem Cells—New Hope for Spinal Cord Injury

### 4.1. Human Embryonic Stem Cells

Human embryonic stem cells (hESC) are derived from the inner cell mass of the preimplantation blastocysts, by removing the trophectoderm cells by immunosurgery [43]. hESC are positive for pluripotent stem cell surface antigens such as stage specific embryonic antigens 3 and 4 (SSEA-3 and SSEA-4), TRA-1-60, TRA-1-81, and express well-known pluripotency-associated genes octamer-binding transcription factor 3/4 (*OCT3*/*4*), sex determining region Y box-containing gene 2 (*SOX2*), and *NANOG* [44,45]. Elevated alkaline phosphatase and telomerase activity are associated with their unlimited proliferative potential [44,45]. These markers are used to verify the successful isolation of a new hESC line and confirm the maintenance of an undifferentiated pluripotent state for established hESC.

**Figure 1:**
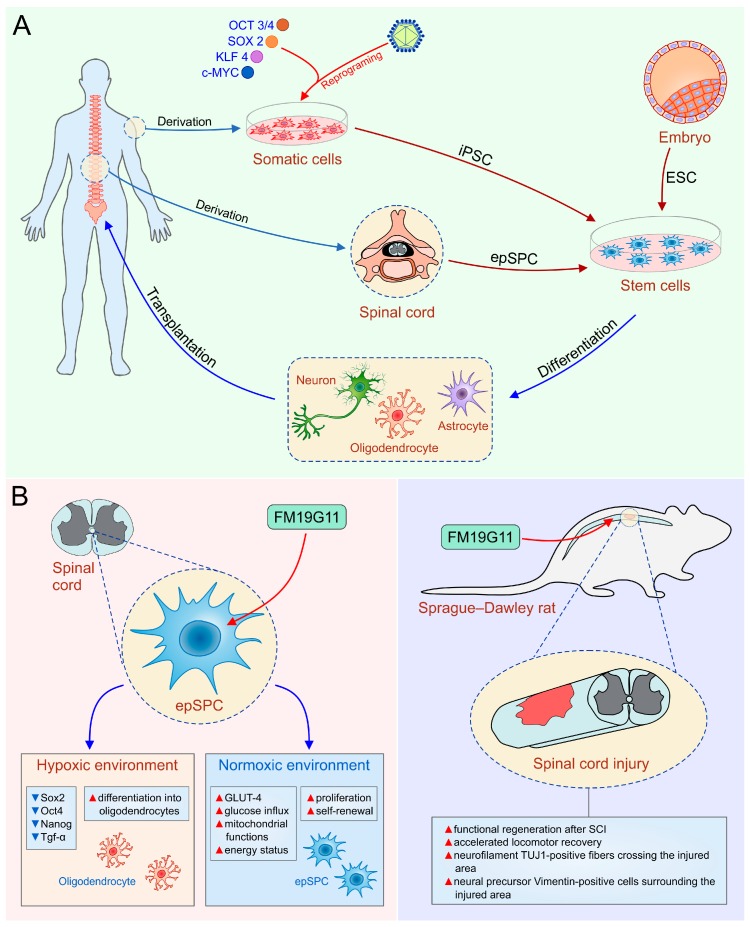
(**A**)Human embryonic stem cells (hESC), induced pluripotent stem cells (iPSC) and ependymal stem/progenitor cells (epSPC) as a promising tool in the therapy of SCI; (**B**) the role of FM19G11, an inhibitor of hypoxia inducible factor (HIFα), to mobilize epSPC. OCT3/4, octamer-binding transcription factor 3/4; SOX2, sex determining region Y box-containing gene 2; KLF4, Krüppel-like factor 4; TGF-α, transforming growth factor-alpha; GLUT-4, glucose transporter type 4.

In addition to remarkable proliferative capacity, hESC exhibit pluripotency both in vitro and in vivo. Because of their ability for differentiation into cells of ectodermal origin such as neuronal and glial cells, hESC are used in many preclinical studies (reviewed in [46]) as a new therapeutic option for SCI (Figure 1A). Several previously published papers have shown that transplantation of hESC-derived oligodendrocyte progenitor cells (OPC) to SCI models resulted in cell survival and clinically relevant recovery of neurological functions with no evidence of harmful effects [47,48,49].

Keirstead and coworkers demonstrated that hESC-derived OPC transplanted seven days after SCI in rats differentiate into mature oligodendrocytes, induce myelin sheath regeneration and significantly improve locomotor function [48]. In contrast, OPC administration ten months after injury, did not manage to improve neurological outcome in injured animals compared with controls, suggesting that first week after SCI is the optimal time point for OPC transplantation [48]. Neural stem cells (NSC) clonally derived from murine embryonic stem cells (dNSCs), without embryoid bodies formation, survive and differentiate into neurons, oligodendrocytes, and astrocytes after injection into the spinal cord lesion one week after SCI in mice. Salewski et al. provided the evidence that transplanted dNSCs have broad spectrum of beneficial neuroregenerative effects associated with enhanced remyelination of damage axons [50]. In addition to differentiation into myelin-forming oligodendrocytes, hESC-derived OPC express neurotrophic factors such as neurite growth-promoting factor 2 (NEGF2), hepatocyte growth factor (HGF), activin A, transforming growth factor-beta 2 (TGF-β2), and brain-derived neurotrophic factor (BDNF), providing significant therapeutic effects in SCI such as neuronal survival and neurite extension [51,52].

In order to increase the yield of defined hESC-derived neural lineages, we optimized in vitro conditions for the differentiation of hESC towards motoneuron progenitors (MP) and OPC using chemically defined mediums without animal components and without feeder cells. This protocol induces conversion of hESC into rosettes and neural tube-like structures with capacity to differentiate into region specific and functional neurons, astrocytes, and oligodendrocytes [53]. For the first time, we achieved controlled differentiation of neural progenitors towards specific type of neuronal cells by stimulating the rosettes with specific signaling factors in vitro [53]. Promising results obtained under in vitro conditions suggest that neuroregenerative potential of hESC-derived OPC and MP should be investigated using an animal model of SCI. Therefore, we used a well-established rat model of complete spinal cord transection, that resemble the pathology of the most severe clinical cases of SCI in humans [54]. Our study showed that transplanted cells OPC and MP survived for at least 4 months, and migrated at least 3 mm away from the site of injury [55]. Main mechanisms of behavioral and electrophysiological improvement after OPC and MP transplantation in SCI were their differentiation into mature oligodendrocytes and neurons and their capacity to produce various neurotrophic factors [55]. Additionally, transplanted OPC and MP triggered Janus kinase/signal transducers and activators of transcription (JAK/STAT) and Notch signaling in the lesion site leading to enhanced astrogliosis [56] indicating that reactive astrocytes in synergy with transplanted cells promote survival and growth of serotonergic and dopaminergic axons [56].

Although the results of preclinical study are promising, there are important issues such as the possibility of immune rejection and the risk of tumor formation after transplantation that should be addressed to achieve successful hESC-based therapy [57].

### 4.2. Induced Pluripotent Stem Cells

Induced pluripotent stem cells (iPSC) were originally obtained by the viral transduction of four transcription factors: *SOX2*, *OCT3/4*, tumor suppressor Krüppel-like factor 4 [*KLF4*], and proto-oncogene *c-MYC* in differentiated somatic cells [58]. The standard viral integrative reprogramming techniques are associated with many risks including insertional mutagenesis, uncontrolled expression of integrated transgenes—downregulation or silencing of the transgenes or tumor formation due to residual reactivation of transgenes, senescence-associated DNA changes, and immunogenicity of iPSC-derived cells [59]. Huge efforts have been devoted toward the development of novel protocols in order to improve quality and efficiency of reprogramming technology and to bring iPSC-derived cells closer to clinic. During the last decade, several studies suggested alternative non-integrative delivery methods for more safety iPSC generation such as use of adenovirus and Sendai virus as well as non-viral-mediated molecular strategies (*Cre-loxP*-mediated recombination, *PiggyBac*-transposition episomal DNA vectors, and direct miRNA transfection) [60,61,62,63,64]. All these technologies provide an opportunity to derive pluripotent cells similar to hESC in terms of morphology, karyotype, and phenotype without destruction of human embryos. The use of patient-specific iPSC for treatment of SCI is particularly attractive, given that they avoid the ethical considerations and immunological rejection of hESC and represent a source of autologous cells that can be differentiated to neural progenitor cells (NPC), neurons, oligodendrocytes, and astrocytes at the same time underlining the integration of transplanted cells into the site of injury as an important issue of successful cell-based therapy (Figure 1A). Several research groups reported that iPSC are capable of generating mature dopaminergic neurons [65,66], motor neurons (MN) [67,68,69], and GABAergic interneurons [70], however, it is known that transplantation of mature neurons is characterized by poor cellular engraftment versus transplantation of neural progenitors. Consequently, transplantation of neural progenitors has been a focus, and seems to be a promising approach for the treatment of SCI. Several groups reported that autologous iPSC derived neural precursor cells (iPSC-NPC) could be efficiently derived [71,72] and used for transplantation into rodents with SCI [73,74]. Transplanted NPC predominantly gave rise to myelin-producing oligodendrocytes, leading to remyelination and improvement of nerve conduction [73]. Moreover, iPSC-NPC migrated long distances, integrated into the spinal cord and differentiated into mature neurons and glia, resulting in synaptic reconstruction and locomotor recovery [75,76,77]. In addition, neurotrophic factors produced by iPSC-NPC, modulate immunopathological events following SCI [78]. Hayashi and colleagues found that iPSC-derived astrocytes injected into the injured rodent spinal cord increased the sensitivity to mechanical stimulus but did not affect locomotor functions [79].

Although patient-specific iPSC are a revolutionary tool which could pave the way to personalized medicine, many issues remain to be improved including reprogramming techniques as well as protocols for targeted differentiation.

### 4.3. Ependymal Stem/Progenitor Cells

It has been demonstrated that OPC, astrocytes, and ependymal cells are the most important dividing cells in the adult spinal cord [80,81,82]. Astrocytes and OPC have the capacity to self-renew, however they cannot give rise to different types of specialized cells, indicating that they are not NSC [83]. Ependymal stem/progenitor cells (epSPC) are adult multipotent stem cells characterized by the ability to differentiate into the both glial cells and neurons [18,56] and can be found around the spinal central canal [4] (Figure 1A, Table 1).

OPC, present in the white and gray matter in the adult CNS, are the main proliferating cell types in the intact spinal cord [80]. Under homeostatic conditions, neural/glial antigen 2 (NG2)—expressing OPC proliferate and differentiate into mature oligodendrocytes maintaining tissue integrity over the lifespan. Following SCI, OPC migrate to the spinal cord lesion sites, and extensively contribute to remyelination [80,81]. The recent studies [84,85] have shown that glial growth factor 2 (GGF2) and ferritin administration promote oligodendrogenesis and improve functional recovery after SCI. Thus, enhancing the endogenous OPC response to injury could be a potential therapeutic approach to manage tissue repair and regeneration without the transplantation of exogenous cells.

It is well known that astrocyte turnover is low in healthy spinal cords, however, these cells respond to SCI by intensive dividing and forming the border of glial scar with fibromeningeal and NG2^+^glia cells [80,86]. Astrocytes in the scar’s periphery inhibit axonal growth in the environment of SCI through secretion of inhibitory molecules such as heparan sulphate proteoglycan, dermatan sulphate proteoglycan, keratan sulphate proteoglycan and chondroitin sulphate proteoglycan (CSPG) [87]. In addition to the extracellular matrix components, semaphorin 3, ephrin-B2 and its receptor EPHB2 and the Slit proteins produced by reactive astrocytes, have largely negative effects on nerve regeneration [87]. Following these observations, several in vivo studies provided the evidence that application of chondroitinase ABC (ChABC) as an individual therapy or in combination with other treatments degraded CSPG in scar tissue, enhanced growth and regeneration of axons, and re-established neural pathways below the lesion (reviewed in [88]). Enzyme-based treatments may provide new opportunities to overcome detrimental effects of glial scar and may offer new hope for success in therapy of SCI.

However, as we mentioned, reactive astrocytes surrounding the spinal cord lesion prevent an excessive inflammatory cell infiltration leading to decrease in immune-mediated damage of spinal cord. Interestingly, during the last two decades, there is growing evidence suggesting novel cellular and molecular mechanisms underlying neuroprotective effect of astrogliosis after SCI. The fact that glial scar is composed of at least two phenotypically and developmentally different populations of astrocytes, may explain a dual role of scar tissue on spinal cord regeneration. Although the most extensive proliferation of epSPC occurs during embryogenesis and the early postnatal period, there are data suggesting slow proliferation rate of epSPC surrounding spinal cord in adulthood [89,90,91]. More importantly, endogenous epSPC are activated 72 h after SCI to migrate from spinal central canal towards the lesion site, divide extensively, and generate astrocytes, as well as a small number of oligodendrocytes [80,82,92,93]. Only small portion of epSPC differentiate toward oligodendrocytes responsible for myelin production, typically located in white matter areas of spinal cord [94]. Most of the epSPC-derived progeny express astrocytic markers [82], however, these reactive astrocytes are glial fibrillary acidic protein (GFAP) negative, invade glial scar center and promote axonal growth and regeneration [80,82,95]. In contrast to astrocytes constituting the glial scar border, epSPC-derived reactive astrocytes actively maintain extracellular homeostasis. In particular, astrocytic uptake of glutamate prevents glutamate-mediated neuronal loss, suggesting that pharmacological stimulation of astrocytes may be a promising therapeutic target for SCI as well as other pathologies involving excitotoxicity. In addition, astrocytes provide significant metabolic support to neurons, regulate extracellular potassium level, and prevent generation of free radicals [96]. The neuroprotective capacity of astrocytes is mediated by wide variety of soluble factors including brain-derived neurotrophic factor, ciliary neurotrophic factor, nerve growth factor (NGF), and basic fibroblast growth factor (FGF-2) and the extracellular matrix molecules laminin and fibronectin [97]. Thus, specific modulation of two reactive astrocyte subpopulations in the injured spinal cord, protective in the core and harmful at the periphery of glial scar, could represent a new regeneration strategy for SCI.

Although the precise mechanism for improved functional recovery after intrathecal administration of epidermal growth factor (EGF) and fibroblast growth factor 2 (FGF-2) is not known [98], these findings suggested that stimulation of resident NSC might be used as a possible new therapeutic approach for SCI treatment. Endogenous cell-based therapeutic approach avoids the risks of exogenous cell transplantation such as risk of tumor formation after engraftment, and possible immunogenicity that requires immunosuppression. Thus, we explored a new chemical entity, FM19G11 as a new pharmacological agent for spinal cord regeneration (Figure 1B). FM19G11, by HIF2α-mediated mechanisms, inhibits the transcriptional and protein expression of pluripotency markers *Sox2*, *Oct4*, *Nanog*, and *Tgf*-*α* in epSPC, leading to increased differentiation of epSPC into oligodendrocytes in a hypoxic environment [99]. However, under normoxic conditions, FM19G11 stimulates glucose intake and mitochondrial functions in epSPC, causing an increased energy status and high proliferation of epSPC (Figure 1B, left panel). Furthermore, we confirmed neuroregenerative properties of FM19G11 using an animal model of SCI [100]. In line with previous findings, paralysis of hind limbs was significantly reversed in FM19G11-treated rats with SCI compared to vehicle treated animals [100] (Figure 1B, right panel). Furthermore, we showed that epSPC isolated from rats with an SCI (epSPCi) display enhanced capability for self-renewal and differentiation toward oligodendrocyte progenitors compared to epSPC from healthy animals [56]. This isconsistent with the fact that the inflammatory environment to which quiescent epSPCare exposed after SCI modulates gene expression profile and induces recruitment of endogenous epSPCto the lesion site [82,92]. In order to delineate the molecular mechanisms responsible for higher regeneration ability of epSPCi after injury, we analyzed the role of Cx50, an ion channel involved in differentiation of stem cells into glial cells within the injured area. In non-pathological conditions, epSPC show high expression levels of Cx50, however, activated epSPCi express low levels of Cx50, indicating adverse effects of Cx50 in spinal cord regeneration [18,101]. An additional molecular mechanism of increased regenerative capacities of epSPCi versus epSPC could be changes in purinergic receptors (P2Y) expression in stem cells. We showed that downregulation of P2Y1 receptor and an upregulation of P2Y4 receptor increases differentiation potential and proliferation ability of epSPCi, respectively [102]. Hence, transplantation of epSPCi or epSPCi-derived oligodendrocyte precursors [OPCi] immediately after spinal cord contusion was a more efficient therapeutic strategy for the locomotor recovery one week after treatment than epSPC or OPC transplantation [56]. Therefore, our studies revealed that transplanted epSPCi differentiated rarely and that beneficial effect of transplanted epSPCi in SCI was primarily based on their release of trophic and immunomodulatory factors that alter function of immune cells [56]. Combination of epSPCi transplantation and local application of FM19G11 reduced glial scar and enhanced generation of oligodendrocyte precursor, however, did not significantly improve the neurological outcome compared to the individual treatments [103]. Therefore, to treat SCI as complex disorder, new hope might be found in a combination of cell transplantation, pharmacotherapy, mobilization of endogenous stem cells and bioscaffolds [103,104].

## 5. Conclusions

Stem cell therapy in SCI provides a clue to solve the challenges which currently used medical procedures cannot treat. Because of their neuroregenerative, neuroprotective and immunomodulatory properties, stem cells are an innovative approach for the therapy of SCI. The presence of NSC in the adult CNS raises the possibility of the modulation of an endogenous regenerative process. Although further investigations are necessary to confirm neurological benefits by adjusting doses and drug administrations, treatments for mobilization of endogenous stem cell population have been considered as a promising therapeutic approach to enhance repair mechanisms in SCI. Additionally, results of preclinical studies indicate that application of stem cell-derived progenitors significantly reduces neurological disability in most severe SCIs. However, the main safety issue regarding pluripotent stem cell-based transplantation is still a lack of efficient protocols to obtain pure cell populations without the presence of unwanted cell types, and undifferentiated hESC/iPSC. It is important to highlight that stem cell transplantation alone is not sufficient to bridge a spinal cord lesion, therefore, a repair strategy based on combination of well-established therapeutic modalities, including surgery and medications, and stem cell-derived neural cells is an extremely attractive option for the treatment of this devastating injuries. Therefore, it is critical to develop new modalities such as directly applied pharmacology and biomaterials that support stem cell survival and provide better tissue integration. Future studies must be focused on resolving issues such as ideal sources of stem cells and safety of stem cell-based therapy with the aim to utterly exploit the promising therapeutic potential of both exogenous and endogenous stem cells in SCI.

## Figures and Tables

**Table 1:** The capacity of engraftment and differentiation, contribution to functional recovery and risk of tumorigenesis of transplanted hESC, iPSC and epSPC in animal model of spinal cord injury Stem cell source.

Stem Cell Source	Differentiation	Engraftment	Contribution to Functional Recovery	Tumorigenesis
hESC	differentiation into neurons and glia [53,64]	hESC-derived OPCs and MPs engraft for at least 4 months in the lesion site [55]	significant improvement of behavioral and electrophysiological, function of injured animals at early time points after SCI [55,56]	risk of teratoma formation [46,57]
iPSC	differentiation into neural progenitor cells, neurons, oligodendrocytes, and astrocytes [72,73,74,75,76,77]	integration for at least 12 weeks after transplantation into injured spinal cord tissue [74]	iPSC-derived cells promote functional recovery in an early SCI model [72,73,74,75,76,77]	more tumorigenic than hESC due to genetic and epigenetic aberrations [46]
epSPC	differentiation into glial cells (oligodendrocytes and astrocytes) and neurons [18,34]	detected 2 months after transplantation [56]	accelerates recovery of motor activity 1 week after injury [56]	low rates of tumorigenesis [4]

hESC, human embryonic stem cells; OPC, oligodendrocyte progenitor cells; MP, motoneuron progenitors; iPSC, induced pluripotent stem cells; epSPC, ependymal stem/progenitor cells; SCI, spinal cord injury.
